# Comparative Safety and Efficacy of Immunosuppressive Regimens Post-kidney Transplant: A Systematic Review

**DOI:** 10.7759/cureus.43903

**Published:** 2023-08-22

**Authors:** Shahid Qayyum, Kamran Shahid

**Affiliations:** 1 Nephrology, Diaverum Dialysis Center, Wadi Al Dawasir, SAU; 2 Internal Medicine/Family Medicine, California Institute of Behavioral Neurosciences & Psychology, Fairfield, USA

**Keywords:** kidney transplantation, sirolimus, everolimus, bleselumab, belatacept, calcineurin inhibitors, transplant nephrology, graft rejection, immunosuppression

## Abstract

Immunosuppressive agents are used post-organ transplant to prevent acute rejection and graft losses. Tacrolimus, the most widely used immunosuppressive agent for kidney transplant recipients, has unfavorable side effects such as new-onset diabetes after transplant, nephrotoxicity, and electrolyte imbalances. Other drug groups such as the mammalian target of rapamycin (mTOR) inhibitors, belatacept, and bleselumab have been used to either substitute calcineurin inhibitors or reduce their exposure. This systematic analysis reviews evidence from randomized controlled trials to compare the safety and efficacy of various immunosuppressive regimens for kidney transplant recipients. An in-depth methodical search was conducted across PubMed, Cochrane Library, and Mendeley. PRISMA 2020 guidelines were followed for this study. Randomized controlled trials comparing varying regimens were included in this study. While there was no difference in safety and efficacy between once-daily and twice-daily tacrolimus, mTOR inhibitors showed to be a viable option for a reduced tacrolimus exposure regimen. Calcineurin inhibitor avoidance and early steroid withdrawal regimens both showed increased rates of rejection. Based on these findings, a regimen containing once-daily tacrolimus and an mTOR inhibitor with or without corticosteroid is a viable immunosuppressive regimen post-kidney transplant. Further trials, especially ones with longer follow-up periods, are needed to explore these regimens' long-term safety and efficacy.

## Introduction and background

Immunosuppression (IS) is used in kidney transplant recipients (KTRs) to reduce the chances of rejection and graft loss and can be categorized as either an induction or maintenance agent. Kidney Disease-Improving Global Outcomes (KDIGO) recommends the use of either lymphocyte-depleting agents such as anti-thymocyte globulin (ATG) or interleukin two (IL-2) receptor antagonist such as basiliximab for induction, which can be started before or at the time of the transplant [1]. For maintenance immunotherapy, the available agents are calcineurin inhibitors (CNIs) such as tacrolimus and cyclosporin, antiproliferative agents such as mycophenolate (mycophenolate mofetil [MMF] or enteric-coated mycophenolate sodium [EC-MPS]), corticosteroids, mammalian target of rapamycin (mTOR) inhibitors such as everolimus and sirolimus, costimulation blocker such as belatacept, and bleselumab (an anti-CD40 monoclonal antibody) [2-4].

Belatacept acts by inhibiting CD28-mediated T-cell costimulation by binding to CD80 and CD86 on the surfaces of antigen-presenting cells [5]. Bleselumab, a human IgG4 anti-CD40 monoclonal antibody, weakens the immune response by inhibiting the interplay of CD40:CD154 between antigen-presenting cells, T-cells, and B-cells [6]. Sirolimus, an mTOR inhibitor, inhibits cytokine production and blocks the cytokine-mediated signal transduction pathway in the T-cell cycle [7]. Everolimus, another mTOR inhibitor, has a similar mechanism to that of sirolimus. It works by blocking the T-cell response by inhibiting growth-driven transduction signals [8]. CNIs dampen the immune response by interfering with T-cell activation, proliferation, and differentiation by inhibiting calcineurin activity, which is needed for the transcription of IL-2 and other cytokines in T-lymphocytes [9].

According to the 2019 Organ Procurement and Transplantation Network Annual Data Report, the most commonly prescribed maintenance IS regimen was a combination of tacrolimus, MMF, and corticosteroids, accounting for approximately 66% of all cases. Approximately 30% of the patients received a combination of tacrolimus and MMF without the use of steroids [10]. CNIs have played a vital role in the reduction in the rate of rejections and graft loss but have undesirable side effects such as nephrotoxicity, hypertension, new-onset diabetes after transplant (NODAT), dyslipidemias, hyperuricemia, and electrolyte imbalances [11].

Efforts have been made to substitute CNIs or reduce their exposure in KTRs using the other available groups. Attempts have also been made to assess the efficacy of either corticosteroid-free or early steroid withdrawal (ESW) regimens. In this systematic review, we will attempt to compare various clinical trials using varying maintenance immunosuppressive regimens in terms of their safety and efficacy in human subjects.

## Review

Method

A methodical literature search was conducted by following the Preferred Reporting Items for Systematic Reviews and Meta-Analyses (PRISMA) criteria. Full-text publications, paid and free, indexed in PubMed, Cochrane Library, and Mendeley were searched from inception to 2023, using the keywords "Kidney transplantation” and “Immunosuppression.” PubMed was searched using Mesh terms "Kidney Transplantation"[Majr] AND "Immunosuppressive Agents"[Majr]. The comprehensive search technique using the three data sources is shown in Table 1.

**Table 1:** Databases used and the search strategy using keywords

Number	Database	Keywords	Search Result
1	PubMed	Kidney transplantation AND Immunosuppression	8,692
2	Cochrane Library	Kidney transplantation AND Immunosuppression	4,035
3	Mendeley	Kidney transplantation AND Immunosuppression	12,486

After the search completion, duplicates were found and removed, and the relevant publications were chosen by inspecting the titles and abstracts. Articles in English (from 2011 to 2023) were included. Only randomized controlled trials (RCTs) were included. Studies on other types of transplants such as liver and pancreas were excluded, and so were studies conducted on animal subjects.

Study Selection

We looked for RCTs that assessed for efficacy and/or safety of immunosuppressive medications post-renal transplant. We excluded all studies that were not available in the English language, animal studies, gray literature, case reports, book chapters, editorials, and systematic reviews. Articles were first screened using the titles and abstracts only, and relevant articles were later screened using the full text.

Data Extraction and Analysis

We extracted data based on authors, year, intervention, gender, percentage of African Americans to study effect based on race, and total sample size. Data were extracted and cross-checked by both authors, and any disputes were solved. A narrative synthesis was performed on the extracted data.

Risk-of-Bias Assessment

Risk-of-bias (ROB) assessment was done using the Revised Cochrane risk-of-bias tool for randomized trials (RoB 2), which uses five domains to judge the quality of the trials. The included domains were ROB arising from the randomization process, ROB due to deviations from the intended interventions, ROB due to missing outcome data, ROB in the measurement of the outcome, and ROB in the selection of the reported result. The traffic light and the summary plot were created using the Risk-of-bias VISualization (robvis) tool [12].

Results

Search Results

**Figure 1:**
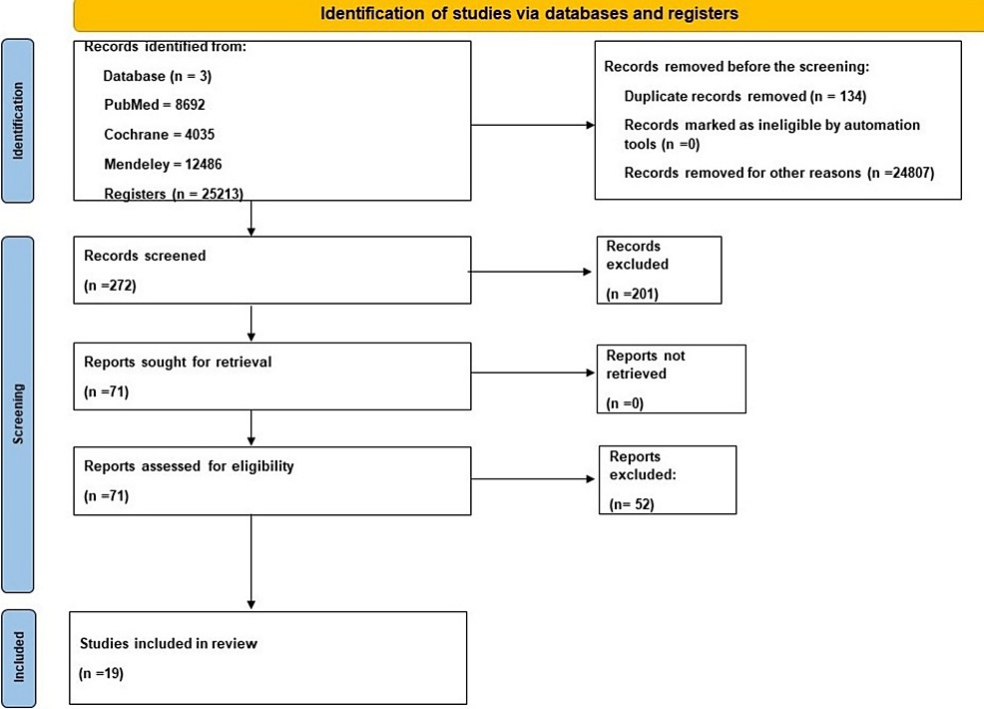
PRISMA flow chart of the literature and the search strategy PRISMA, Preferred Reporting Items for Systematic Reviews and Meta-Analyses

A thorough search from three databases yielded a total of 25,213 articles; 134 duplicates were removed and a further 24,807 articles were excluded because they did not meet the inclusion criteria. A total of 272 articles were screened using titles and abstracts, out of which 71 remaining articles were assessed for eligibility; only 19 were included in this review. Figure 1 shows the PRISMA flowchart of the literature and the search strategy of the studies [13].

ROB of Clinical Trials

**Figure 2:**
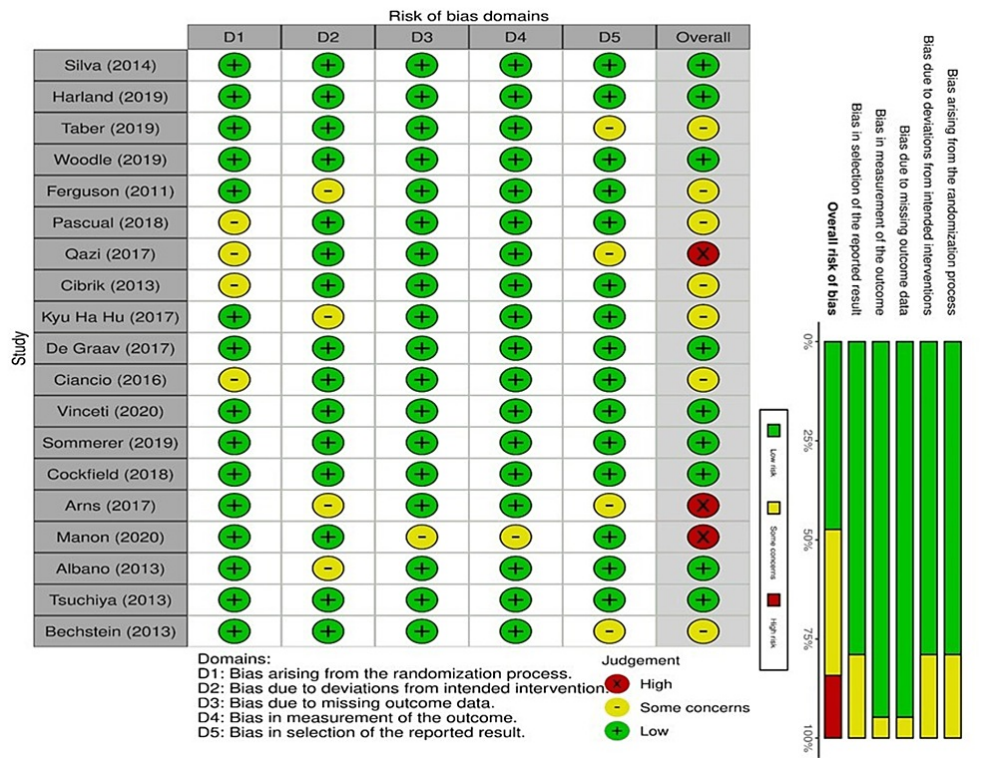
Traffic light and summary plot for included trials Silva et al. (2014) [14]; Harland et al. (2019) [4]; Taber et al. (2019) [15]; Woodle et al. (2019) [16]; Ferguson et al. (2011) [17]; Pascual et al. (2018) [18]; Qazi et al. (2017) [19]; Cibrik et al. (2013) [20]; Kyu Ha Hu et al. (2017) [21]; De Graav et al. (2017) [22]; Ciancio et al. (2016) [23]; Vinceti et al. (2020) [24]; Sommerer et al. (2019) [25]; Cockfield et al. (2018) [26]; Arns et al. (2017) [27]; Manon et al. (2020) [28]; Albano et al. (2013) [29]; Tsuchiya et al. (2013) [30]; Bechstein et al. (2013) [31]

Quality appraisal was thoroughly done for all included RCTs. The traffic light and the summary plot for the articles are shown in Figure 2.

Characteristics of Included Studies

All included studies were published between 2011 and 2023. The following data were extracted from each article: author, study design, sample size, gender, intervention, and percentage of African Americans. A summary of the characteristics of included studies and descriptions of intervention are described in Table 2.

**Table 2:** Characteristics of included studies ACEi, angiotensin-converting enzyme inhibitor; ARBs, angiotensin receptor blocker; BD, twice daily; CNI, calcineurin inhibitor; CS, corticosteroids; ESW, early steroid withdrawal; F, female; IR, immediate release; M, male; QD, once daily; rATG, rabbit anti-thymocyte globulin; TAC, tacrolimus; XL, extended release; Y, yes

Author Name	Design	Intervention	Gender	Number of African Americans (%)	Sample Size	Outcome
Silva et al. (2014) [14]	Long-term follow-up report of phase III, comparative, noninferiority study	Arm 1: Astagraf XL QD and mycophenolate and CS; arm 2: Prograf BD and mycophenolate and CS; arm 3: Neoral BD and mycophenolate and CS	M: 404; F: 234	128 (20.1)	Total = 638 Arm 1: 226 (214 dosed) Arm 2: 219 (212 dosed) Arm 3: 223 (212 dosed	Safety: Y Efficacy: Y
Harland et al. (2019) [4]	Phase II, open-label, randomized, noninferiority study	Arm 1: IR-TAC 0.1 mg/kg/day and mycophenolate and CS; arm 2: bleselumab 200 mg and mycophenolate and CS; arm 3: bleselumab 200 mg and IR-TAC 0.1 mg/kg/day and CS	M: 93; F: 46	30 (21.6)	Total = 139 Arm 1: 49 Arm 2: 46 Arm 3: 44	Safety: Y Efficacy: Y
Taber et al. (2019) [15]	Randomized, open-label, parallel clinical trial	Arm 1: IR-TAC and mycophenolate and prednisone; arm 2: transition to low-dose TAC and everolimus and prednisone at three months post-transplant	M: 46; F: 14	34 (56.7)	Total = 60 Arm 1: 30 Arm 2: 30	Safety: Y Efficacy: Y
Woodle et al. (2019) [16]	Randomized, open-label, clinical trial	Arm 1: Alemtuzumab and belatacept and ESW; arm 2: rATG and belatacept and ESW; arm 3: rATG and tacrolimus and ESW	M: 212; F: 104	42 (13.3)	Total = 316 Arm 1: 107 Arm 2: 104 Arm 3: 105	Safety: Y Efficacy: Y
Ferguson et al. (2011) [17]	Randomized, open-label exploratory study	Arm 1: belatacept and mycophenolate with ESW; arm 2: belatacept and sirolimus with ESW; arm 3: TAC and mycophenolate with ESW	M: 67; F: 22	16 (18.0)	Total = 93 (4 not transplanted) Arm 1: 33 Arm 2: 26 Arm 3: 30	Safety: Y Efficacy: Y
Pascual et al. (2018) [18]	Randomized, noninferiority clinical trial	Arm 1: everolimus and low-dose CNI; arm 2: mycophenolate and standard dose CNI	M: 1,417; F: 620	78 (3.8)	Total = 2,037 Arm 1: 1,022 Arm 2: 1,015	Safety: Y Efficacy: Y
Qazi et al. (2017) [19]	Randomized, open-label, noninferiority study	Arm 1: everolimus and low-dose TAC; arm 2: mycophenolate and standard dose TAC	M: 407; F: 203	144 (23.6)	Total = 613 (3 not dosed) Arm 1: 306 Arm 2: 304	Safety: Y Efficacy: Y
Cibrik et al. (2013) [20]	Phase III b, randomized, open-label clinical trial	Arm 1: everolimus (trough concentration of 3-8 ng/mL) and low-dose cyclosporin; arm 2: everolimus (trough concentration of 6-12 ng/mL) and low-dose cyclosporin; arm 3: mycophenolate and standard dose cyclosporin	M: 557; F: 276		Total = 833 Arm 1: 277 Arm 2: 279 Arm 3: 277	Safety: Y Efficacy: Y
Kyu Ha Hu et al. (2017) [21]	Randomized, open-label, noninferiority clinical trial	Arm 1: extended-release TAC and low-dose sirolimus and CS; arm 2: extended-release TAC and low-dose mycophenolate and CS	M: 110; F: 41		Total = 151 Arm 1: 76 Arm 2: 75	Safety: Y Efficacy: Y
De Graav et al. (2017) [22]	Randomized, open-label, parallel-group clinical trial	Arm 1: belatacept and mycophenolate and CS; arm 2: TAC and mycophenolate and CS	M: 30; F: 10	4 (10)	Total = 40 Arm 1: 20 Arm 2: 20	Safety: Y Efficacy: Y
Ciancio et al. (2016) [23]	Randomized, open-label clinical trial	Arm 1: TAC and everolimus with ESW; arm 2: TAC and mycophenolate with ESW	M: 23; F: 7	7 (23.3)	Total = 30 Arm 1= 15 Arm 2= 15	Safety: Y Efficacy: Y
Vinceti et al. (2020) [24]	Phase I b, randomized, double-blind, placebo-controlled, parallel-group trial	Arm 1: placebo; arm 2: bleselumab 50 mg; Arm 3: bleselumab 100 mg; arm 4: bleselumab 200 mg; arm 5: bleselumab 500 mg	M: 34; F: 11	2 (4.4)	Total: 45 Arm 1: 8 Arm 2: 10 Arm 3: 9 Arm 4: 10 Arm 5: 8	Safety: Y Efficacy: Y
Sommerer et al. (2019) [25]	Randomized, open-label clinical trial	Arm 1: everolimus and TAC; arm 2: everolimus and cyclosporine; arm 3: mycophenolate and TAC	M: 411; F: 201		Total = 612 Arm 1: 208 Arm 2: 199 Arm 3: 205	Safety: Y Efficacy: Y
Cockfield et al. (2018) [26]	Randomized, open-label controlled trial	Arm 1: low TAC and ACEi/ARB + mycophenolate + CS; arm 2: low TAC and other antihypertensive therapy + mycophenolate + CS; arm 3: standard dose TAC and ACEi/ARB + mycophenolate + CS; arm 4: standard dose TAC and other antihypertensive therapy + mycophenolate + CS	M: 191; F: 90		Total = 281 Arm 1: 71 Arm 2: 69 Arm 3: 71 Arm 4: 70	Safety: Y Efficacy: Y
Arns et al. (2017) [27]	Randomized, open-label, parallel group clinical trial	Arm 1: generic TAC (TacHexal); arm 2: branded TAC (Prograf)	M: 58; F: 15		Total = 73 Arm 1: 35 Arm 2: 38	Safety: Y Efficacy: Y
Manon et al. (2020) [28]	Randomized, open-label, prospective trials	Arm 1: rATG (induction) + mycophenolate and TAC; arm 2: rATG (induction) + mycophenolate and belatacept; arm 3: basiliximab (induction) + mycophenolate and TAC and belatacept	M: 48; F: 21	34 (49.3)	Total = 69 Arm 1: 29 Arm 2: 29 Arm 3: 11	Safety: Y Efficacy: Y
Albano et al. (2013) [29]	Randomized, open-label, parallel group clinical trial	Arm 1: TAC BD 0.2 mg/kg/day and mycophenolate and CS (tapered) over 24 weeks; arm 2: TAC QD 0.2 mg/kg/day and mycophenolate and CS (tapered) over 24 weeks; arm 3: TAC QD 0.3 mg/kg/day and mycophenolate and CS (tapered) over 24 weeks; arm 4: TAC QD 0.2 mg/kg/day and mycophenolate and basiliximab and CS only preoperatively	M: 806; F: 392	39 (3.3)	Total = 1198 Arm 1: 309 Arm 2: 302 Arm 3: 304 Arm 4: 283	Safety: Y Efficacy: Y
Tsuchiya et al. (2013) [30]	Randomized, open-label comparative study	Arm 1: TAC QD and mycophenolate; arm 2: TAC BD and mycophenolate	M: 69; F: 33		Total = 102 Arm 1: 50 Arm 2: 52	Safety: Y Efficacy: Y
Bechstein et al. (2013) [31]	Randomized, open-label trial	Arm 1: sirolimus and low-dose TAC; arm 2: sirolimus and standard dose TAC	M: 83; F: 45		Total = 128 Arm 1: 63 Arm 2: 65	Safety: Y Efficacy: Y

Discussion

**Figure 3:**
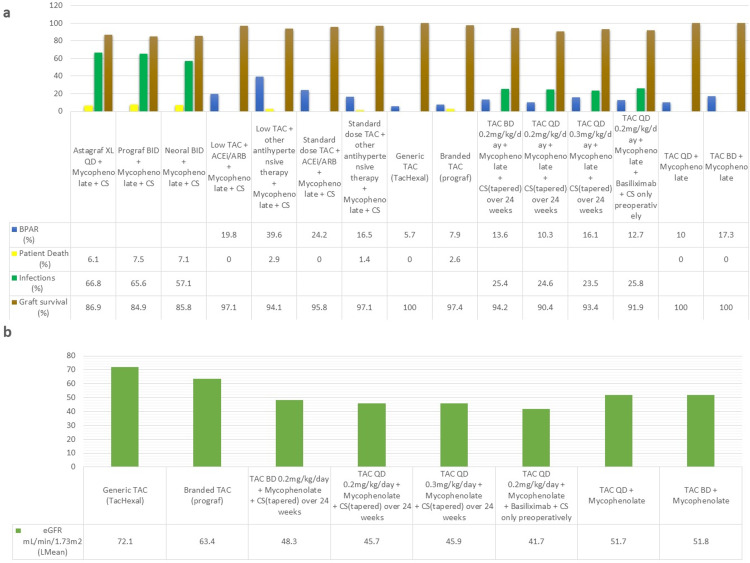
Safety and efficacy outcome data for tacrolimus-based regimens a=rate of rejection, patient deaths, and infections; b=eGFR mL/min/1.73m^2^ (least mean) ACEi, angiotensin-converting enzyme inhibitor; ARB, angiotensin receptor blocker; BID, twice daily; BPAR, biopsy-proven acute rejection; CS, corticosteroids; eGFR, estimated glomerular filtration rate; Lmean, least mean; QD, once daily; TAC, tacrolimus; XL, extended release

CNIs have a narrow therapeutic index, and blood levels need to be monitored in order to minimize nephrotoxicity [32]. Medication non-compliance can lead to graft rejection and loss [33]. To increase the rate of compliance, a once-daily dose of tacrolimus was developed. Silva et al. [14] conducted a phase III clinical trial comparing once-daily (QD) extended-release tacrolimus (brand name: Astagraf XL) and twice-daily (BD) tacrolimus (brand name: Prograf) with cyclosporin BD. All three arms received mycophenolate and corticosteroids. The group receiving Prograf had the highest rate percentage of graft loss (15.1%) and patient death (7.5%), with an infection rate of 65.6%. Graft loss with Astagraf XL was 13.1%, with a 6.1% rate of patient deaths and a 66.8% rate of infections. Overall, Astagraf XL showed a benefit in renal function compared to the cyclosporin group, but no significant difference was observed compared to Prograf, and there was a higher incidence of NODAT in tacrolimus-based groups. The OSAKA trial [29] consisted of four arms comparing tacrolimus BID and QD. In addition to different tacrolimus dosing, the first three groups received mycophenolate and corticosteroids tapered over 24 weeks, and the last group received mycophenolate, basiliximab, and corticosteroids only preoperatively. The rate of acute rejection was the lowest in the tacrolimus QD at 0.2 mg/kg/day (10.3%) followed by tacrolimus BD at 0.2 mg/kg/day (13.6%), and it was the highest in tacrolimus QD at 0.3 mg/kg/day (16.1%), showing that higher starting dose is not needed. The graft loss was highest in tacrolimus QD at 0.2 mg/kg/day (9.6%), and the renal function was the highest in the tacrolimus BD group (48.3 mL/min/1.73m^2^). The infection rates showed no significant difference between all four groups. This trial concluded that increasing the started dose provided no added benefit for efficacy. Arns et al. [27] conducted a randomized trial comparing generic Tacrolimus (TacHexal) with branded Tacrolimus (Prograf). Although there was a mean difference (MD) of 9.1 mL/min/1.73m^2^ in the estimated glomerular filtration rate (eGFR) in favor of TacHexal, this trial concluded that there were no differences in efficacy between the two groups after the application of the last observation carried forward analysis. There were no deaths or graft losses in TacHexal group, but one (2.6%) patient lost the graft and one (2.6%) patient died due to an unknown cause in the Prograf group. Cockfield et al. [26] conducted a four-arm trial comparing standard and low-dose prolonged release tacrolimus. Each group received either an angiotensin-converting enzyme inhibitor (ACEi) or angiotensin receptor blockers (ARBs), or other antihypertensive medications. This trial showed the beneficial effects of adding ACEi/ARBs with low-dose tacrolimus, with 19.8% of patients experiencing T-cell mediated rejection (TCMR) in the ACEi/ARB group as compared to 39.6% with other antihypertensive medications. When comparing low-dose tacrolimus to the standard dose, there was a reduced number of TCMRs in patients receiving the standard dose, showing that reducing the dose of tacrolimus has its drawbacks. The trial conducted by Tsuchiya et al. [30] showed that there was no added benefit in reducing the exposure of tacrolimus and that there is an increased incidence of biopsy-proven acute rejection (BPAR) when the exposure is reduced. These trials showed that there was no significant difference in efficacy and safety between brands and no added efficacy benefits to increasing exposure, but the addition of ACEi/ARBs has an added benefit in efficacy. While there were comparable efficacies in QD and BD dosing, QD dosing might be a better option since it provides easier dosing for patients. Figures 3a, 3b show the safety and efficacy outcome data.

**Figure 4:**
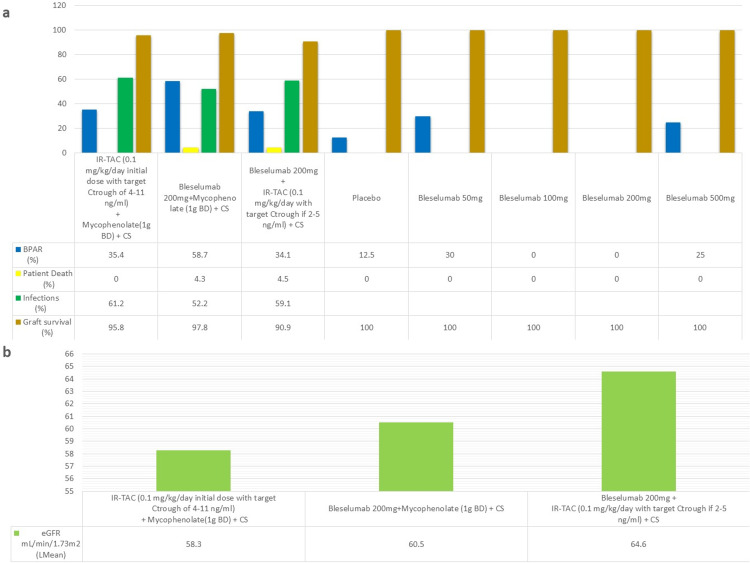
Safety and efficacy of bleselumab-based regimens a=rate of rejection, graft survival, patient death, and infections; b=eGFR mL/min/1.73m^2^ (least mean) BPAR, biopsy-proven acute rejection; IR, immediate release; TAC, tacrolimus; Ctrough, trough concentration; BD, twice daily; CS, corticosteroids; eGFR, estimated glomerular filtration rate; Lmean, least mean

A phase I b study for bleselumab [24] found that the treatment with all tested doses was well tolerated with no significant immediate or long-term side effects. There were no reported graft losses in all five groups. There were no reported BPAR in 100 mg and 200 mg doses. Overall, 12.5% of patients experienced BPAR in the placebo group, 30% in the 50 mg group, and 25% in the 500 mg group. Harland et al. [4] conducted a phase II trial comparing the standard of care (immediate-release tacrolimus and MMF) with bleselumab plus MMF and bleselumab plus immediate-release (IR) tacrolimus. This trial found that the bleselumab plus MMF group had the highest rate of BPAR and that bleselumab plus IR-tacrolimus was not inferior to the standard of care for the prevention of BPAR. The authors concluded that there was no statistically significant difference between the groups in terms of eGFR, graft loss, and patient deaths. Figures 4a, 4b show the safety and efficacy outcome data.

**Figure 5:**
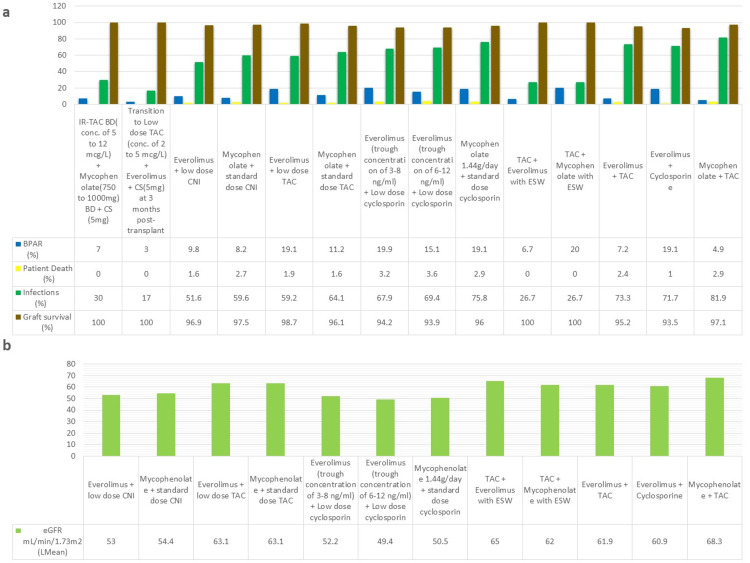
Safety and efficacy data from trials with everolimus-based regimens a=rate of rejection, graft survival, patient death, and infections; b=eGFR mL/min/1.73m^2^ (least mean) BD, twice daily; BPAR, biopsy-proven acute rejection; CNI, calcineurin inhibitor; eGFR, estimated glomerular filtration rate; ESW, early steroid withdrawal; IR, immediate release; Lmean, least mean; TAC, tacrolimus

Taber et al. [15] conducted a trial to assess the influence of conversion to everolimus and low-dose tacrolimus compared with staying on the standard care regimen of IR-tacrolimus, mycophenolate, and prednisone. The trial showed that switching to everolimus has comparable efficacy in the rate of BPAR and lower rates of opportunistic infections. There were no reported deaths or graft losses in either group, making the transition a viable option for post-transplant IS. A trial conducted by Sommerer et al. [25] also showed comparable efficacy in terms of BPAR in groups receiving everolimus plus tacrolimus and mycophenolate plus tacrolimus. This study also provided evidence that the group receiving everolimus had the lowest infection rates, especially BK virus (BKV) and cytomegalovirus (CMV) infections. The eGFR was the highest in the group receiving tacrolimus and mycophenolate. A trial comparing tacrolimus/everolimus and tacrolimus/EC-MPS [23] showed an equal rate of infections in both groups and had comparable eGFR with a lower rate of BPAR in the tacrolimus/everolimus group. Pascual et al. [18] also showed that the group receiving everolimus had lower rates of infection with BKV and CMV, but results were comparable for BPAR, graft losses, patient deaths, and eGFR. The trial by Qazi et al. [19] once again showed that patients receiving everolimus had lower rates of CMV and BKV infections and comparable eGFR and patient death. The group receiving everolimus with a low dose of tacrolimus had a higher rate of BPAR but fewer graft losses. The trial by Cibrik et al. [20] was a clinical trial comparing everolimus (trough concentration of either 3-8 or 6-12 ng/mL) plus low-dose cyclosporin A (CsA) and mycophenolate and standard dose CsA. The trial showed that everolimus is a viable option to reduce exposure to CsA as it provided comparable eGFR and BPAR rates, while groups receiving everolimus had lower rates of CMV and BKV infections. Everolimus was generally well tolerated. These trials showed us that everolimus is a practical option for reducing exposure to CNIs and that it offers increased protection against infections, especially by BKV and CMV while having a similar efficacy to the standard dose CNIs. Figures 5a, 5b show the safety and efficacy data from the trials.

**Figure 6:**
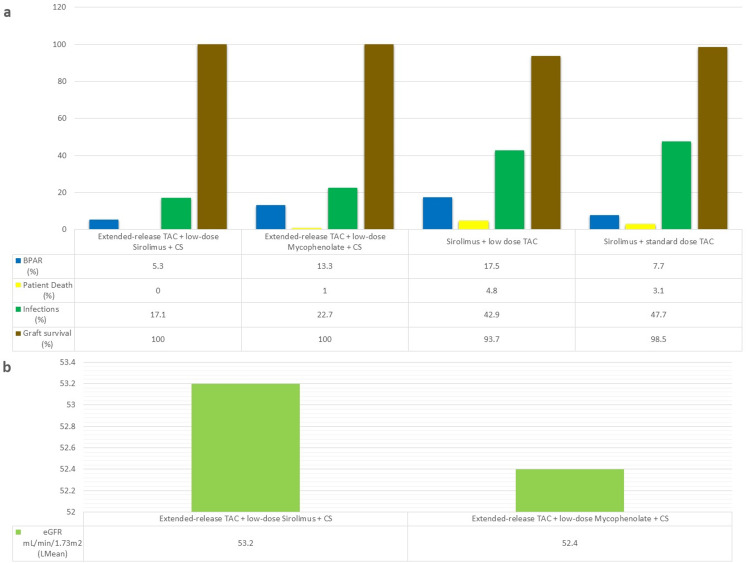
Safety and efficacy data from trials with sirolimus-based regimens a=rate of rejection, graft survival, patient death, and infections; b=eGFR mL/min/1.73m^2^ (least mean) BPAR, biopsy-proven acute rejection; CS, corticosteroids; eGFR, estimated glomerular filtration rate; Lmean, least mean; TAC, tacrolimus

A trial comparing the combination of sirolimus with either low-dose tacrolimus or standard-dose tacrolimus [31] showed that the low-dose tacrolimus group had favorable protection against acute rejection, and renal function in this group was significantly improved as compared to standard-dose tacrolimus. Sirolimus was generally well-tolerated and had no unexpected adverse effects, and the reduction in tacrolimus exposure may have the added benefit of reducing CNI-mediated adverse effects. A trial comparing low-dose sirolimus and low-dose MMF [21] found the extended-release tacrolimus plus sirolimus to be comparable, in both safety and efficacy, with extended-release tacrolimus plus MMF. In addition, the sirolimus group had a significantly lower incidence of CMV and BKV infections. Low-dose sirolimus can allow minimization of tacrolimus exposure and help reduce CNI-mediated adverse effects and preserve renal function. Figures 6a, 6b show the safety and efficacy outcome data.

**Figure 7:**
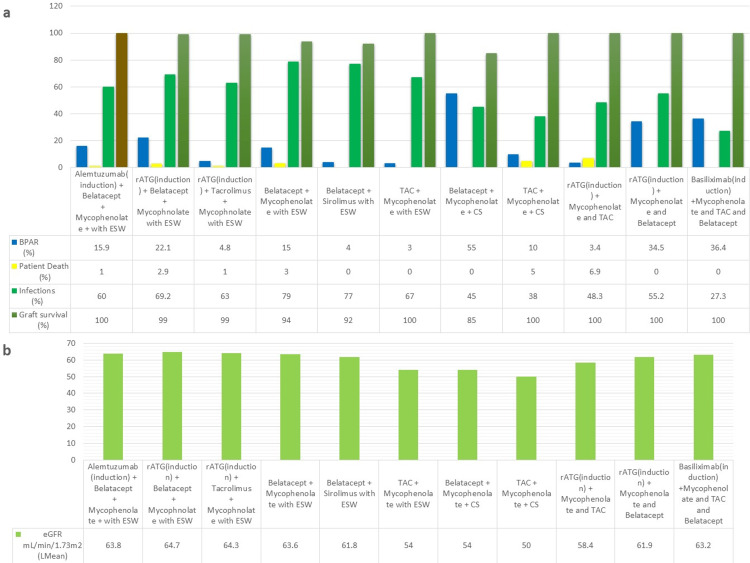
Safety and efficacy data from trials with belatacept-based regimens a=rate of rejection, graft survival, patient death, and infections; b=eGFR mL/min/1.73m^2^ (least mean) BPAR, biopsy-proven acute rejection; CS, corticosteroids; eGFR, estimated glomerular filtration rate; ESW, early steroid withdrawal; rATG, rabbit anti-thymocyte globulin; Lmean, least mean; TAC, tacrolimus

Ferguson et al. [17] found that belatacept-based regimens had improved renal functions compared to tacrolimus. This trial had ESW and concluded that belatacept-based regimens and ESW has comparable safety and efficacy profile. A study comparing belatacept with tacrolimus [22] found that belatacept is not as effective as tacrolimus for the prevention of acute rejection. While the eGFR between the two groups was comparable, the group receiving belatacept had a staggering 55% incidence of BPAR compared to the 10% incidence in the tacrolimus group. A trial by Woodle et al. [16] compared not only tacrolimus and belatacept but also two T cell-depleting agents, rabbit anti-thymocyte globulin (rATG), and alemtuzumab. The rate of rejection was higher in both belatacept-based groups, and eGFR values were comparable in all groups. The results of alemtuzumab and rATG were comparable. This trial also showed a low incidence of post-transplant lymphoproliferative disorder (PTLD), which has been a safety concern with belatacept. Although the rejection rates were higher in the belatacept groups, the belatacept-based regimen with T-cell depleting agent and ESW can provide increased graft survival rates. The trial by Mannon et al. [28] once again showed a higher incidence of rejection in belatacept-based groups. One benefit belatacept has over tacrolimus is patient adherence to IS because of regular and monitored infusion visits. Belatacept while being able to provide an avenue for reduced CNI exposure and ESW showed poor protective capabilities against acute rejection. Figures 7a, 7b show the safety and efficacy data from the trials.

Limitations

African Americans have twice the risk of graft loss when compared to Caucasians with similar mortality risks. At the one-year mark, acute rejection rates in African Americans is 6.7% compared to 7.6% in Caucasians [34]. This disparity between races was one of the limitations of this systematic review as some included studies did not mention the distribution of races, which could have introduced a bias in the efficacy comparison. Furthermore, there were inconsistencies in reporting data such as age, history of any previous transplantation, pre-existing diabetes, and other chronic diseases, and whether the donor was living or deceased. Lastly, some of the studies we included had moderate-to-high concerns for bias. Despite these limitations, efforts were made to make the comparison as unbiased as possible.

## Conclusions

In conclusion, this systematic review shows that increasing the exposure to tacrolimus has little to no added benefit and that lowering exposure without the addition of another immunosuppressive agent increased the rates of acute rejection and graft losses. There was no difference in safety and efficacy between QD and BD tacrolimus. Everolimus and sirolimus are both viable options when considering a dose reduction of tacrolimus as the rate of rejection was comparable, renal function was improved, and the rate of CMV and BKV infections were lower in groups treated with everolimus or sirolimus. Bleselumab, when given with IR-tacrolimus, is comparable to standard of care in both safety and efficacy. Avoidance of CNI and ESW using belatacept showed higher rates of rejection and graft losses. Based on the given findings, a regimen comprising once-daily tacrolimus and an mTOR inhibitor with or without corticosteroids is a viable option for IS for KTRs.
